# Supplementary material for: Trends and patterns of dual use of combustible tobacco and e‐cigarettes among adults in England: A population study, 2016–2024
Source: Addiction. 2025 Jan 22;120(4):608–19. doi: 10.1111/add.16734 (PMC11907328; doi:10.1111/add.16734)
Supplement: Supplementary file 1 — Figure S1. Prevalence of dual use of smoking and vaping vs. exclusive smoking in England, 2016 to 2024, overall and by sociodemographic characteristics. Figure S2. Age‐specific trends in the overall prevalence of dual use of smoking and vaping in England, July 2016 to April 2024. Figure S3. Patterns of dual use of smoking and vaping in England, by user characteristics. Figure S4. Trends in different patterns of dual use of smoking and vaping in England, 2016 to 2024 – including dual users with missing data on vaping frequency. Figure S5. Trends in different patterns of dual use of smoking and vaping among smokers and dual users in England, 2016 to 2024. [file ADD-120-608-s001.pdf]

Items assessing vaping status and frequency

The following questions assessed vaping status. For each question, interviewers were prompted to probe fully, asking 'Which others?' until the participant responded that they did not use any other of these products.

Participants who reported current smoking were asked:

1. Which, if any, of the following are you currently using to help you cut down the amount you smoke?
    a) Nicotine gum
    b) Nicotine replacement lozenges\tablets
    c) Nicotine replacement inhaler
    d) Nicotine replacement nasal spray
    e) Nicotine patch
    f) Electronic cigarette
    g) Nicotine mouthspray
    h) Heat-not-burn cigarette (e.g. iQOS, heatsticks)
    i) Juul
    j) Tobacco-free nicotine pouch/pod or 'white pouches' that you place on your gum (e.g., Zyn, On!, Nordic Spirit, Velo, Lyft, Skruf)
    k) Other (specify)

2. Do you regularly use any of the following in situations when you are not allowed to smoke?
    a) Nicotine gum
    b) Nicotine lozenge
    c) Nicotine patch
    d) Nicotine inhaler\inhalator
    e) Another nicotine product
    f) Electronic cigarette
    g) Nicotine mouthspray
    h) Heat-not-burn cigarette (e.g. iQOS, heatsticks)
    i) Juul
    j) Tobacco-free nicotine pouch/pod or 'white pouches' that you place on your gum (e.g., Zyn, On!, Nordic Spirit, Velo, Lyft, Skruf)
    k) Other (specify)

Participants who reported past-year smoking (i.e., current smoking or having quit in the past year) were asked:

- Can I check, are you using any of the following either to help you stop smoking, to help you cut down or for any other reason at all?
    a) Nicotine gum
    b) Nicotine lozenge
    c) Nicotine patch
    d) Nicotine inhaler\inhalator
    e) Another nicotine product

f) Electronic cigarette
g) Nicotine mouthspray
h) Heat-not-burn cigarette (e.g. iQOS, heatsticks)
i) Juul
j) Other (specify)

Participants who reported that they quit smoking more than a year ago or had never regularly smoked were asked:

- Can I check, are you using any of the following?
    a) Nicotine gum
    b) Nicotine lozenge
    c) Nicotine patch
    d) Nicotine inhaler\inhalator
    e) Another nicotine product
    f) Electronic cigarette
    g) Nicotine mouthspray
    h) Heat-not-burn cigarette (e.g. iQOS, heatsticks)
    i) Juul
    j) Tobacco-free nicotine pouch/pod or 'white pouches' that you place on your gum (e.g., Zyn, On!, Nordic Spirit, Velo, Lyft, Skruf)
    k) Other (specify)

To assess frequency of use, those who report using one or more of these products in response to any of these questions were then asked:

- How many times per day on average do you use your nicotine replacement product or products?
    a) 1
    b) 2
    c) 3-4
    d) 5-7
    e) 8-11
    f) 12+
    g) Not every day but at least once a week
    h) Not every day and less than once a week
    i) Don’t know

Participants were not given a definition of ‘nicotine replacement product or products’, but this question immediately followed those assessing current product use, described above.

The full questionnaire is available at https://smokinginengland.info/resources/sts-documents.

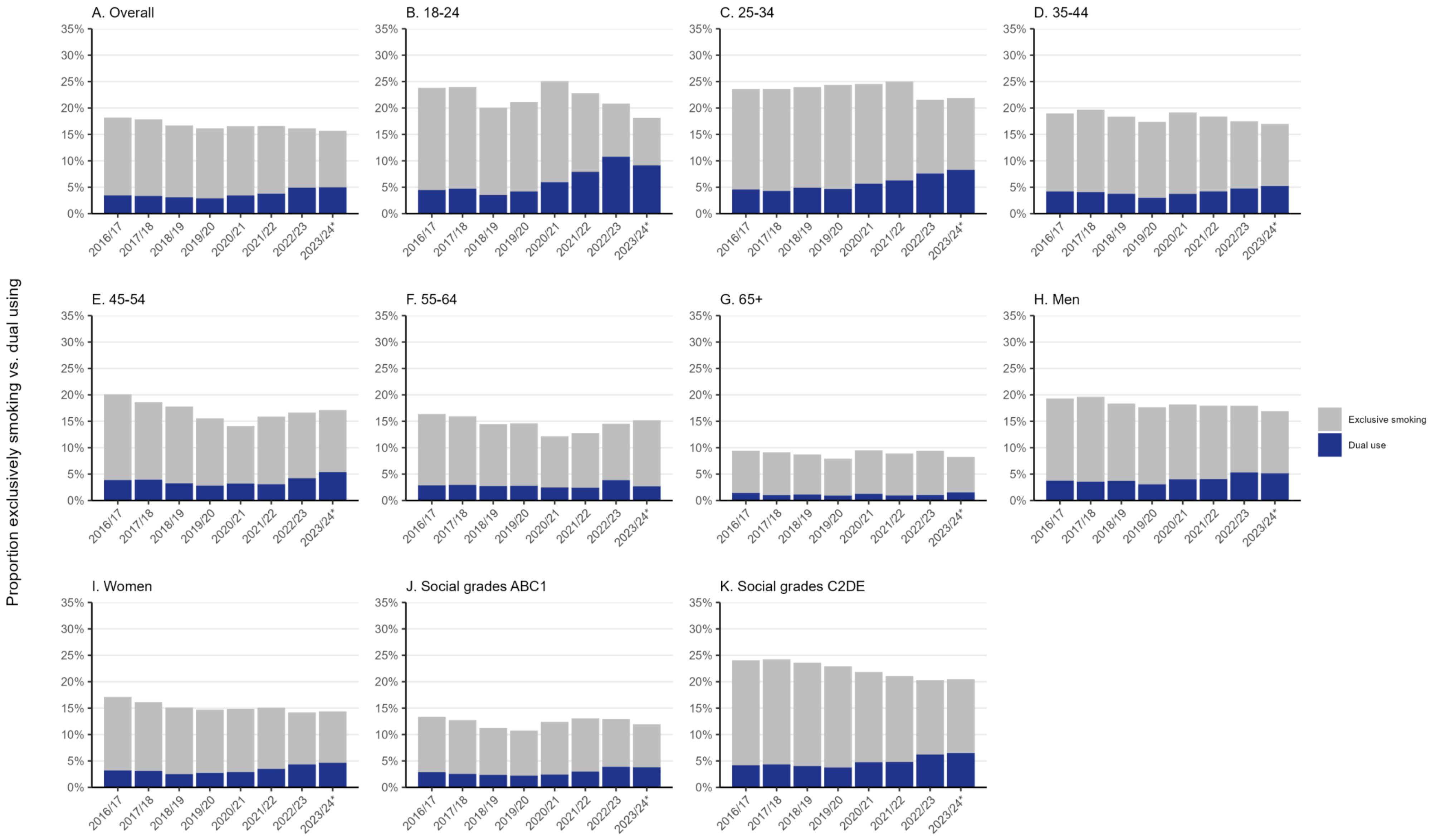

Figure S1. Prevalence of dual use of smoking and vaping vs. exclusive smoking in England, 2016 to 2024, overall and by sociodemographic characteristics.

Proportion of adults in England who report dual use vs. exclusive smoking, aggregated by survey year (July-June), (A) overall and by age (B-G), gender (H-I), and occupational social grade (J-K). ABC1 = more advantaged, C2DE = less advantaged. *2023/24 only includes data up to April.

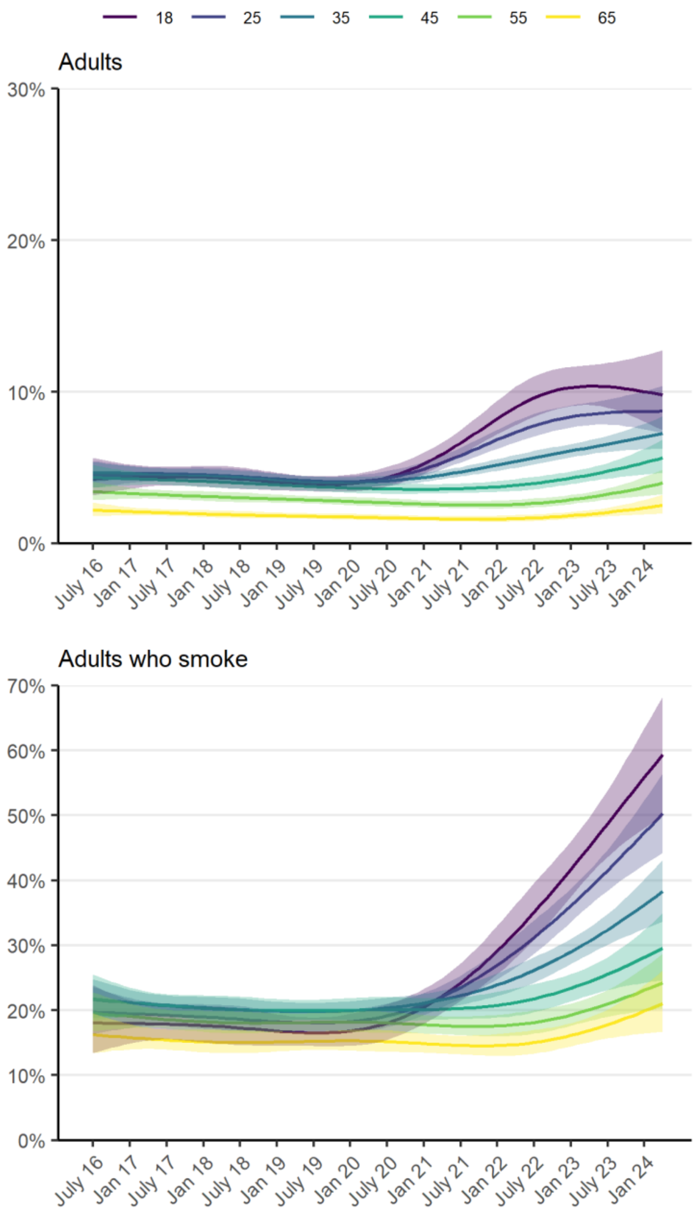

Figure S2. Age-specific trends in the overall prevalence of dual use of smoking and vaping in England, July 2016 to April 2024.

Lines represent the modelled weighted proportion by monthly survey wave and age (modelled non-linearly using restricted cubic splines with five and three knots, respectively). Shaded bands represent 95% confidence intervals. Sample sizes and modelled estimates of prevalence in the first and last months in the time series are reported in **Table 1**.

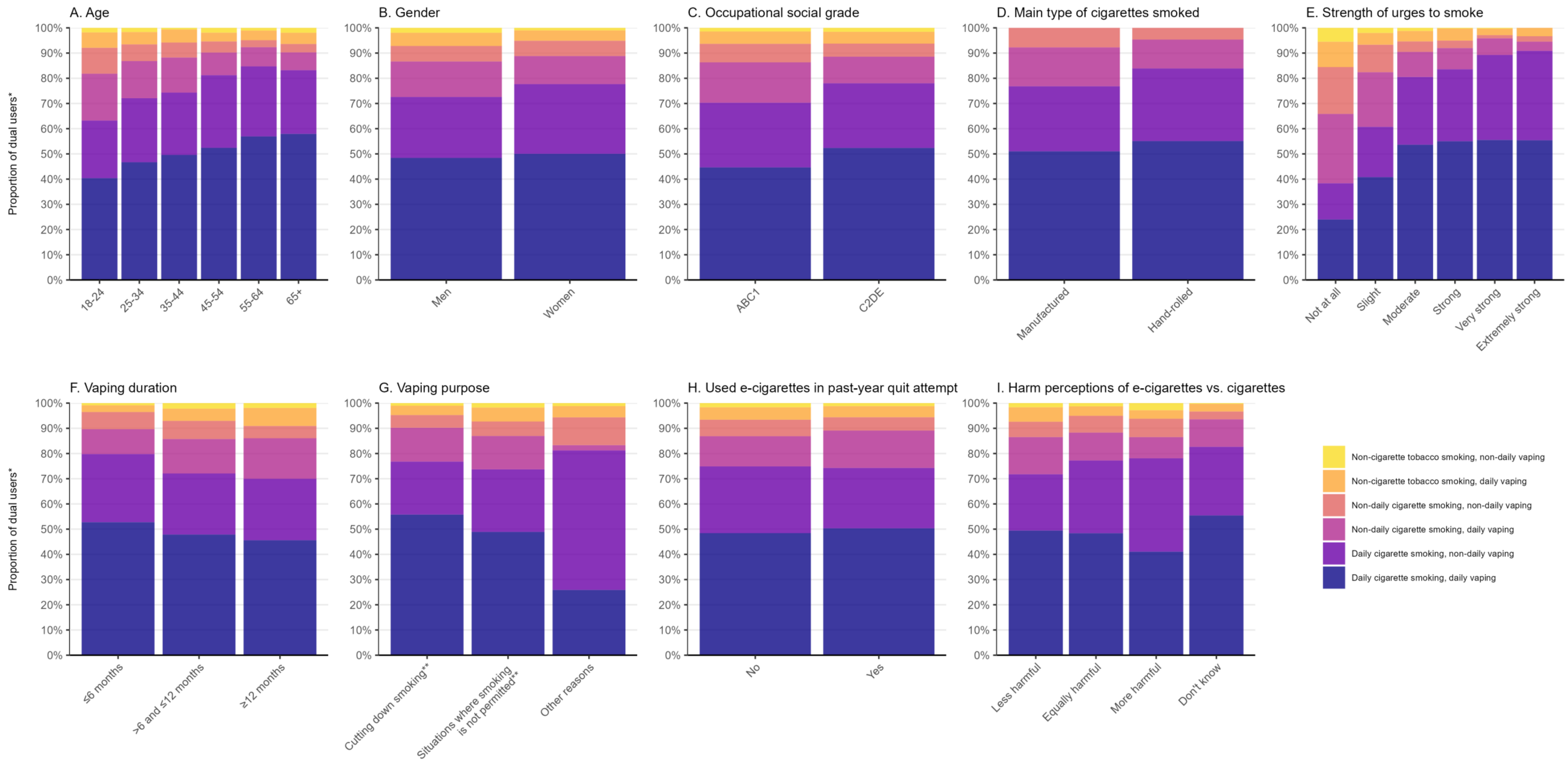

Figure S3. Patterns of dual use of smoking and vaping in England, by user characteristics.

Proportion of dual users who reported each pattern of dual use, by sociodemographic, smoking, and vaping characteristics and harm perceptions of e-cigarettes vs. cigarettes. *Sample excludes dual users who reported that they did not know how frequently they vaped. **These purposes were not mutually exclusive.

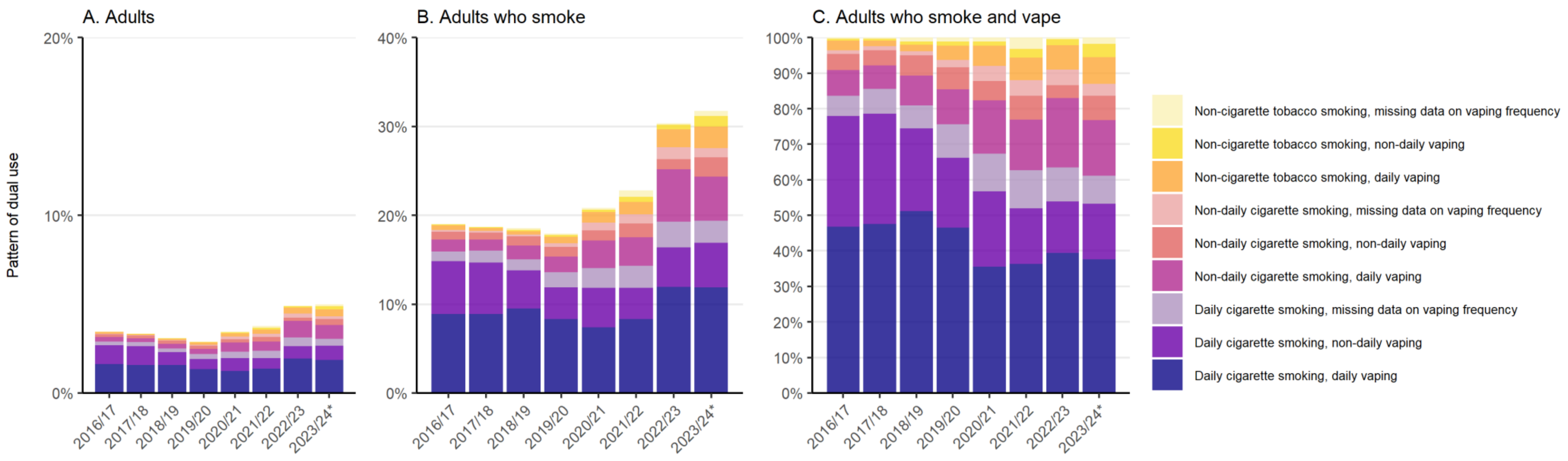

Figure S4. Trends in different patterns of dual use of smoking and vaping in England, 2016 to 2024 – including dual users with missing data on vaping frequency.

Proportion of (A) adults, (B) adults who smoke, and (C) adults who smoke and vape who reported each pattern of dual use, aggregated by survey year (July-June), including dual users who reported that they did not know how frequently they vaped. **2023/24 only includes data up to April.

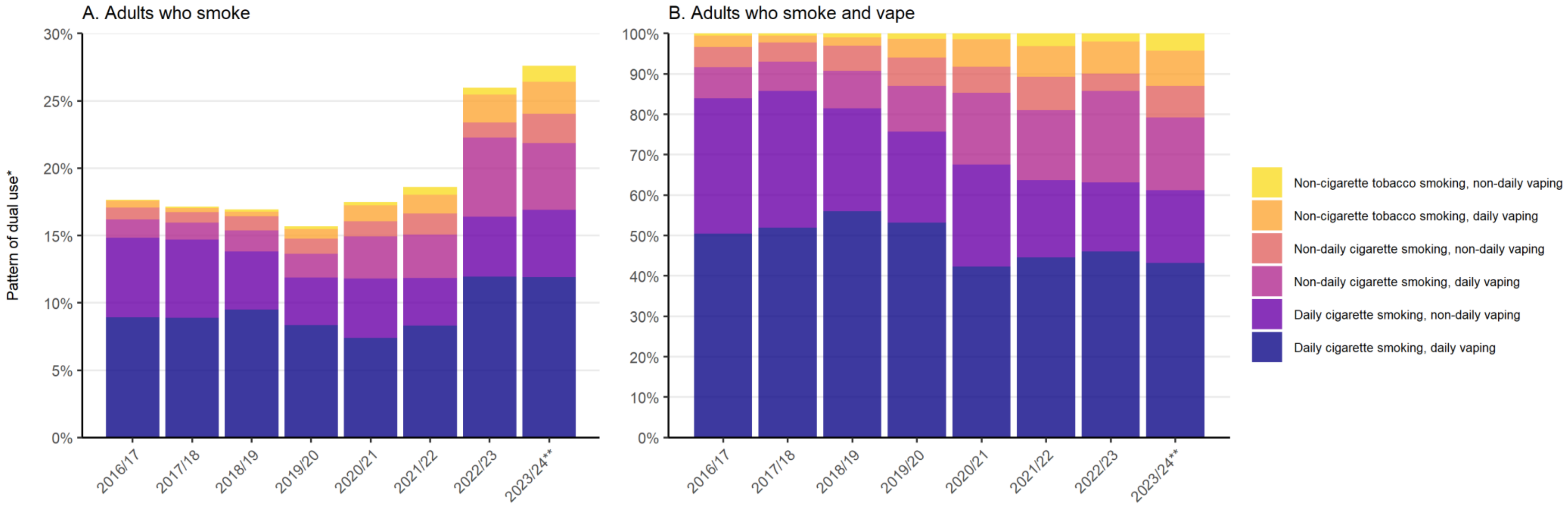

Figure S5. Trends in different patterns of dual use of smoking and vaping among smokers and dual users in England, 2016 to 2024.

Proportion of (A) adults who smoke and (B) adults who smoke and vape who reported each pattern of dual use, aggregated by survey year (July-June). * Data are not shown for dual users who reported that they did not know how frequently they vaped; **Figure S3** shows data including these participants. **2023/24 only includes data up to April.
